# Twenty years of waterborne and related disease reports in Florida, USA

**DOI:** 10.1016/j.onehlt.2021.100294

**Published:** 2021-07-20

**Authors:** Kelly Rhoden, Jose Alonso, Meg Carmona, Michelle Pham, Amber N. Barnes

**Affiliations:** Department of Public Health, University of North Florida, Jacksonville, FL, USA

**Keywords:** One health, Waterborne disease, Vector-borne disease, Water toxins, Zoonoses

## Abstract

Florida represents a unique challenge for preventing and responding to infectious disease associated with water. This study cataloged the prevalence of reportable waterborne and water-related disease within Florida residents over the last twenty years and identified relationships between confirmed cases by location and additional risk factors. Data was collected through FLHealthCHARTS for confirmed cases between January 1, 1999 and December 31, 2019. Case records were compiled and analyzed by year, county, pathogen name and disease category, patient age, and where the infection was acquired. During this time, 218,707 cases of water-related disease were recorded with 214,745 due to waterborne disease, 3255 cases of water-related vector-borne disease, and 707 cases caused by a water-based toxin. Children aged 0–4 and the elderly demonstrated a higher proportion of waterborne disease while 45–49 year olds had increased rates of water-based toxins and water-related vector-borne disease. Most cases were reported in the southeast region. Across the state, opportunities for water contact have led to high rates of water-related infectious disease. Public health initiatives and response efforts should target the pathogens of greatest impact for each region, largely zoonotic waterborne diseases, using a One Health approach.

## Highlights

• Over 200,000 cases of water-related disease have been reported to the Florida Department of Health over the last 20 years
• Most reported disease is due to waterborne pathogens followed by water-related vector-borne disease and water-based toxins
• Salmonellosis makes up the largest reported water-related disease burden for Florida
• Children and seniors have higher risk for waterborne disease; adults have higher risk for toxins and vector-borne disease
• Water disease prevention and response must use a One Health model for collaboration with human and animal health providers

## 1 Introduction

Florida is primarily surrounded by ocean with an expansive coastline and inland freshwater body systems contributing to the state's economy through tourism and recreation, fishing and seafood industries, and transportation and global shipping [1,2]. However, water-based infections can threaten the health and safety of not only people and animals, but also state revenue critical for education, public health, and building and maintaining infrastructure [[3], [4], [5]]. Illness can occur after exposure to a waterborne pathogen, a water-related vector-borne disease, or a water-based toxin or toxin-producer. Transmission of infectious agents through water contact can lead to adverse health effects such as gastrointestinal illness/diarrheal disease, respiratory distress, reproductive and fertility problems, neurological disorders, and even death [6].

Waterborne pathogens are a leading cause of diarrheal-related morbidity and mortality worldwide [7]. Many waterborne diseases are zoonotic and are transmitted when animal or human waste is not properly collected, stored and treated before being released into the shared environment, such as a nearby water source [8]. Agricultural and storm runoff can transfer these organisms across multiple media such as soil, water, sand, and aerosolized particles to rest in locations where humans or animals may become exposed and infected [[8], [9], [10]]. Contaminated water can lead to illness when humans or animals drink, breathe, or come in contact with infectious agents or toxins through their skin, eyes, ears, or other mucous membranes [11].

Water-related vector-borne diseases are spread by insects that depend on water for their life cycle and propagation. A common vector in Florida is mosquitoes, which can transfer disease-producing microorganisms such as parasites, viruses, and bacteria from one infected host to another, whether those hosts be human or animal. There are at least 80 mosquito species circulating throughout the state, five of which feed on humans and can transmit disease [12]. Most Florida counties have a tax-funded mosquito control program with an estimated savings of over $200 for every dollar spent in mosquito research [13].

Infections from water-based toxins and toxin-producers occur primarily through direct contact with, or consumption of, contaminated water and aquatic organisms [14]. Humans can experience symptoms of poisoning after consuming seafood or shellfish that contain water-based toxins [15]. Toxins can accumulate in saltwater, freshwater, and brackish water systems causing Harmful Algal Blooms (HABs) but may also bioaccumulate within aquatic food webs, infecting fish, shore birds, marine mammals, and other species [16]. Depending upon the toxin or toxin-producer, exposure may also occur through the respiratory tract. Humans can suffer respiratory distress and asthma attacks after inhaling aerosolized toxic algal particles [17]. Several water-based toxins can also be fatal to animals who inhale these particles [16].

In Florida, water-related illnesses make up a large portion of the public health disease burden with 137 outbreaks reported to the National Outbreak Reporting System (NORS) from 1999 to 2017 [18]. Yet regional and historical trends have not been analyzed by pathogen category, year, or location. The primary objective of this study is to determine which waterborne diseases, water-based toxins, and water-related vector-borne diseases have been reported within the state of Florida over the last twenty years and identify associated case characteristics. This data will support policy-makers as they consider resource allocation for disease surveillance, case reporting, public health campaigns, and community prevention efforts using a One Health approach.

## 2 Methods

### 2.1 Data collection and search strategy

The Florida Department of Health (FDOH) maintains a list of over 60 reportable diseases and conditions and the local health department must be notified of any new suspect, probable or confirmed case [19,20]. For this study, publicly accessible data on confirmed cases of reportable waterborne diseases, water-based toxins, and water-related vector-borne diseases was queried and exported between March and June 2020 from websites managed by the Florida Department of Health (Table 1). These included the Reportable Diseases Frequency Reports and Florida HealthCHARTS [21]. These databases compile de-identified county health department data from the internal FDOH reporting system known as Merlin. The Merlin tool is based on reportable disease information received by the Florida Department of Health as mandated under Section 381.0031, Florida Statutes, and Rule 64D-3.029, Florida Administrative Code.

**Table 1:** Total counts of confirmed cases of waterborne disease, water-related vector-borne disease, and water-based toxins in Florida, USA from 1999 to 2019 by highest and lowest counties and region.

	Counties^a^ with highest case counts					Counties^a^ with lowest case counts					Florida region^b^							Total Cases
	MD	BRO	PAL	DUV	HIL	LIB	LAF	GLA	MAD	CAL	1	2	3	4	5	6	7	
**Waterborne diseases**																		214,745
Salmonellosis	12,237	8385	7446	6864	5453	51	36	61	72	89	7483	3747	15,796	17,955	21,959	12,014	28,538	107,492
Campylobacteriosis	4479	2429	2078	1483	1502	12	35	19	16	17	13,755	901	3470	4810	3878	3883	9084	27,401
Shigellosis	3978	2706	1422	2076	1891	4	4	14	18	10	1777	811	3657	4966	4814	1945	8192	26,162
Giardiasis	6257	1922	1677	1260	1593	13	13	7	19	20	944	981	3170	4018	4226	2577	9956	25,872
Hepatitis A	1284	714	493	177	784	1	0	4	3	2	177	128	628	3111	2174	970	2536	9724
Cryptosporidiosis	610	603	363	521	573	2	12	1	8	5	272	310	1194	1598	1104	693	1593	6764
Legionellosis	415	416	402	228	240	4	0	0	0	0	117	63	440	888	1003	684	1246	4441
Vibriosis (Vibrio species not *Vibrio cholerae* type O1)	186	167	198	144	162	1	2	3	2	5	348	171	317	522	423	389	596	2766
*Escherichia coli* infection, Shiga toxin producing	342	176	160	85	169	0	3	0	2	1	87	80	234	418	414	305	681	2219
Cyclosporiasis	106	91	162	84	102	0	0	0	0	0	51	38	242	287	272	236	362	1488
Typhoid fever (*Salmonella* serotype Typhi)	79	52	39	9	20	0	0	0	0	0	6	3	22	35	35	29	172	302
Hepatitis E	9	3	2	2	7	0	0	0	0	0	1	0	3	15	9	5	14	47
Cholera (*Vibrio cholerae* type O1)	12	6	2	0	0	0	0	0	0	0	1	0	0	2	5	4	20	32
Leptospirosis	5	0	0	0	4	0	0	0	0	0	0	0	4	5	2	0	6	17
Amebic encephalitis	1	1	1	0	1	0	0	1	0	0	0	1	2	1	3	2	3	12
Tularemia	0	0	1	0	0	0	0	0	0	0	0	0	0	2	1	0	1	4
Melioidosis	0	1	0	0	0	0	0	0	0	0	0	0	0	0	1	0	1	2
Poliomyelitis	0	0	0	0	0	0	0	0	0	0	0	0	0	0	0	0	0	0
**Water-related vector-borne disease**^c^																		3255
Malaria	327	219	145	92	115	0	0	0	0	0	44	37	149	215	265	112	700	1522
Dengue	19	131	80	0	1	0	0	0	0	0	77	25	50	45	160	394	311	1062
West Nile virus	30	8	7	78	7	0	2	0	2	2	121	21	107	37	22	27	49	384
Chikungunya virus	40	30	32	4	22	0	0	0	0	0	4	2	8	46	64	14	103	241
Eastern equine encephalitis	1	0	0	2	3	0	0	0	0	0	6	9	5	6	2	1	1	30
California serogroup virus disease	1	1	1	0	3	0	0	0	0	0	0	1	1	4	0	0	3	9
St. Louis encephalitis	0	0	0	2	0	0	0	0	0	0	1	1	2	0	0	3	0	7
Arboviral diseases not otherwise listed	0	0	0	0	0	0	0	0	0	0	0	0	0	0	0	0	0	0
Venezuelan equine encephalitis	0	0	0	0	0	0	0	0	0	0	0	0	0	0	0	0	0	0
Viral hemorrhagic fevers	0	0	0	0	0	0	0	0	0	0	0	0	0	0	0	0	0	0
Yellow fever	0	0	0	0	0	0	0	0	0	0	0	0	0	0	0	0	0	0
**Water-based toxins**																		707
Ciguatera fish poisoning	302	65	93	11	7	0	0	0	0	0	7	4	40	14	78	54	485	682
Neurotoxic shellfish poisoning	3	2	0	0	3	0	0	0	0	0	0	0	3	5	2	9	5	24
Saxitoxin poisoning (paralytic shellfish poisoning)	0	0	0	0	0	0	0	0	0	0	0	0	0	0	1	0	0	1

a MD = Miami-Dade; BRO = Broward; PAL = Palm Beach; DUV = Duval; HIL = Hillsborough; LIB = Liberty; LAF = Lafayette; GLA = Glades; MAD = Madison; CAL = Calhoun.

b Region 1 = Bay, Calhoun, Escambia, Gulf, Holmes, Jackson, Okaloosa, Santa Rosa, Walton, and Washington counties; Region 2 = Columbia, Dixie, Franklin, Gadsden, Hamilton, Jefferson, Lafayette, Leon, Liberty, Madison, Suwanee, Taylor, and Wakulla counties; Region 3 = Alachua, Baker, Bradford, Clay, Duval, Flagler, Gilchrist, Levy, Marion, Nassau, Putnam, St. John's, and Union counties; Region 4 = Citrus, Hardee, Hernando, Hillsborough, Pasco, Pinellas, Polk, and Sumter counties; Region 5 = Brevard, Indian River, Lake, Martin, Orange, Osceola, St. Lucie, Seminole, and Volusia counties; Region 6 = Charlotte, Collier, Desoto, Glades, Henry, Highlands, Lee, Manatee, Okeechobee, and Sarasota counties; and Region 7 = Broward, Miami-Dade, Monroe, and Palm Beach counties.

c County level data for confirmed cases of Zika fever is not available.

Confirmed cases were limited to reporting dates between January 1st, 1999 and December 31st, 2019, as recorded in the Florida Health Department's online databases. Case reports that fell outside of this date range were excluded, regardless of illness onset date. No probable cases were considered for this analysis. Numbers extracted from Florida HealthCHARTS are confirmed diagnostic cases and are unlikely to be revised. All confirmed cases were included if they were recorded in the FDOH Reportable Disease Frequency Report, regardless of whether the illness was: a) acquired in Florida, b) acquired in the United States but not in Florida, or c) acquired outside of the United States or the location of acquisition is unknown. All 67 counties in Florida were included in the analysis and case frequency by year and reporting county was recorded and analyzed. For the purpose of this study, only infectious diseases were considered and chronic conditions or poisonings of heavy metals were excluded.

Publicly assessable data on confirmed cases of Zika virus during this time frame was not available and this disease was removed from analysis. Each disease was separated into its appropriate category based on primary mode of transmission. Additional reportable diseases that did not fall under the category of waterborne diseases, water-based toxins, and water-related vector-borne diseases were excluded.

The age of reported cases were available for all selected water-related pathogens through the Reportable Diseases Frequency Report database with the exception of dengue virus. Age data for dengue arbovirus was unavailable from the public databases and was omitted. Case reports were included from all ages of the remaining water-related pathogens, ranging from 0 to 85 years and older. For this analysis, age categories were grouped by five year spans (i.e. 0–4) with the eldest category made into 70 and older.

### 2.2 Data analysis

State regions were defined using the same classifications set forth by the Florida Department of Emergency Management (Table 1 footnote) [22]. Region 1 is the western panhandle and region 2 is the central panhandle. Region 3 consists of northeastern central Florida while region 4 is the greater Tampa area, including the central western coast. Region 5 is greater Orlando, including the central eastern coast. Region 6 is southwestern Florida and region 7 is the greater Miami-Dade area and the Florida Keys.

Case frequency data was analyzed for 32 reportable diseases from 1999 through 2019 across the state of Florida. Descriptive analysis was conducted on select variables of interest such as case age, disease, location of residence, reporting year, and where the disease was acquired. Trends were examined related to increasing or decreasing individual case counts, regional disease burden, and categorical disease frequencies. Output was generated using Microsoft Excel (Microsoft Office 365, Excel Version 2007, 2016) and ArcMap 10.6® (ESRI, Redlands, CA, 2018).

## 3 Results

**Fig. 1:**
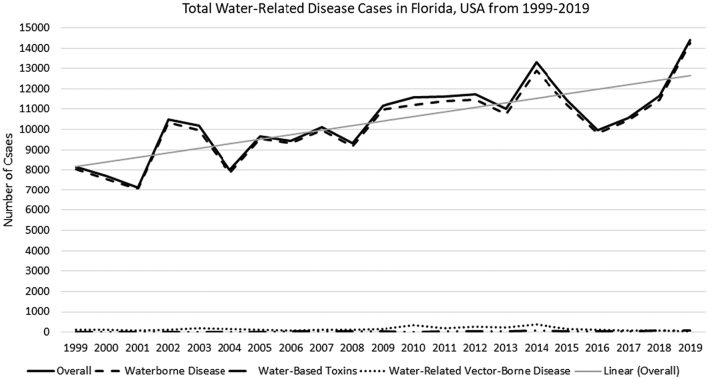
Confirmed cases of waterborne disease, water-related vector-borne disease, and water-based toxins in Florida, USA between 1999 and 2019.

Over the past 20 years, the frequency of cases of water-related disease has been increasing throughout the state of Florida (Fig. 1). Between 1999 and 2019, over 200,000 confirmed cases of water-related reportable diseases were identified across all counties. The year 2019 had the highest number of case reports statewide (n = 14,387) and 2001 had the lowest (n = 7164 cases). The largest uptick of reported cases occurred between 2001 and 2002, with the addition of 3333 cases. Overall there were more confirmed cases of waterborne disease when compared to water-related vector-borne disease and water-based toxins.

The waterborne disease, water-related vector-borne disease and water-based toxin case reports correlate to Florida regions (Table 1). Of the reportable diseases analyzed in this study, waterborne pathogens made up the highest burden of infections for Florida residents. Salmonellosis was reported most often in all regions of the state, with a total count of 107,492 confirmed cases between 1999 and 2019. Following this, the prevalent waterborne pathogens reported were campylobacteriosis (n = 27,401), shigellosis (n = 26,162), giardiasis (n = 25,872) and hepatitis A (n = 9724). The pathogens reported the least in Florida during this time were leptospirosis (n = 17), amebic encephalitis (n = 12), tularemia (n = 4) and melioidosis (n = 2).

Between the years 1999 to 2019, the order of the regions with the most to least cases were as follows: Region 7 (n = 64,658), region 5 (n = 40,917), region 4 (n = 39, 005), region 3 (n = 29,544), region 1 (n = 25,279 cases), region 6 (24,350), and region 2 (n = 7334; Table 1). Three out of the five counties that reported the highest case counts statewide were located in region 7 (Miami-Dade, Broward, and Palm Beach). Miami-Dade county alone contributed almost half, or 48%, of the reportable disease in region 7 (n = 30,723). The next highest counties for state case reports were Duval (region 3) and Hillsborough (region 4), with 13,122 and 12,662 cases respectively. The five counties with the lowest overall case counts for all diseases included Liberty, Lafayette, Glades, Madison, and Calhoun. Liberty county contributed 88 confirmed cases to the overall reportable disease frequency.

**Fig. 2:**
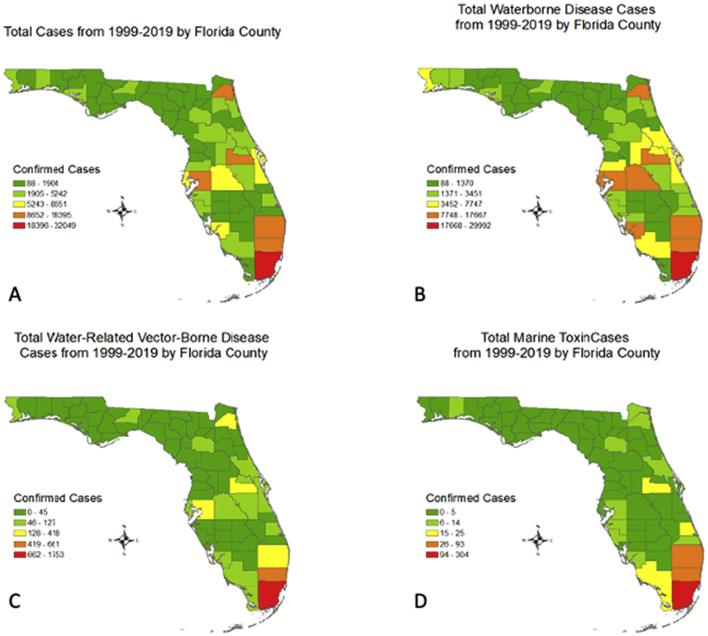
Choropleth maps demonstrating incidence of confirmed cases by Florida county from 1999 to 2019: A) All reported cases combined; B) Cases of waterborne disease only; C) Cases of water-related vector-borne disease only; and D) Cases of water-based toxins only. Map created in ArcMap 10.6 (ESRI, Redlands, CA); no copyrighted material was used. ESRI, Environmental Systems Research Institute.

Region 7 had the most confirmed cases of waterborne disease, water-related vector-borne disease, and water-based toxins equating to 30% of the total 218,707 state cases between 1999 and 2019. Case density mapping is illustrated for each Florida county by total water-related reports and by disease category (Fig. 2). Miami-Dade county exhibited the highest number of total cases (Fig. 2A) and reports for each disease category such as waterborne disease (Fig. 2B), water-related vector-borne diseases (Fig. 2C), and water-based toxins (Fig. 2D). Broward county also demonstrated consistently high numbers of cases throughout each category of disease. A similar case density was observed for Palm Beach county for waterborne disease and water-based toxins only.

**Fig. 3:**
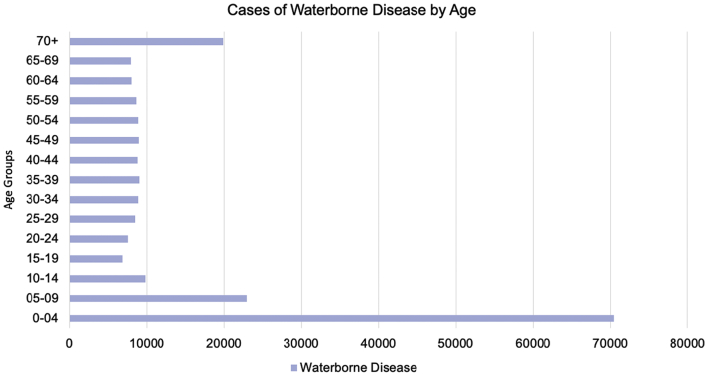
Confirmed cases of Florida waterborne disease cases between 1999 and 2019 by age group.

The age group most impacted by waterborne diseases was children between 0 and 4 years (Fig. 3). This group contracted waterborne diseases at more than double the rate of any other age group during the last two decades (n = 70,544). The age ranges with the second and third highest confirmed case count of waterborne disease was 5–9 year olds (n = 23,013) followed by adults 70 years and older (n = 20,127).

**Fig. 4:**
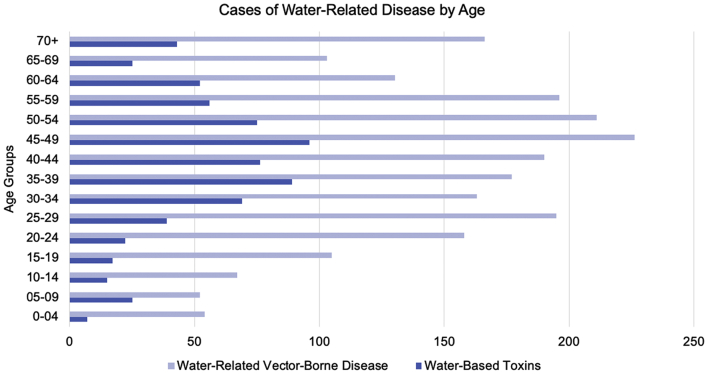
Confirmed cases of Florida water-based toxins and water-related vector-borne disease cases between 1999 and 2019 by age group.

Water-based toxins and water-based vector-borne diseases affected Floridians at much lower rates than waterborne disease. Within both categories of disease, the age group with the greatest disease burden was 45–49-year olds, who accounted for 226 cases of water-related vector-borne disease and 96 cases of water-based toxins (Fig. 4). Case data for confirmed Zika virus was inaccessible and omitted.

**Fig. 5:**
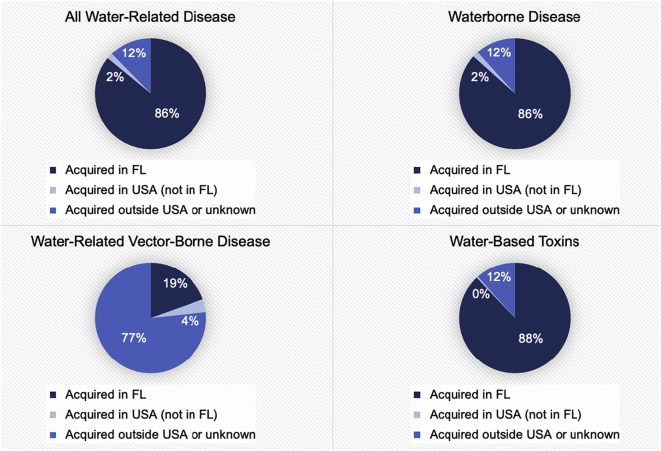
Categories of acquisition for confirmed cases of Florida water-related diseases between 1999 and 2019.

Overall, most cases of water-related disease in residents were acquired in Florida (n = 187,088; Fig. 5). The exception was in the category of water-based vector-borne disease, wherein the majority of exposure occurred outside of the United States or at an unknown location (n = 1682). A large number (n = 25,050) of cases of waterborne disease were also acquired outside of the Unites States or from an unknown region.

## 4 Discussion

This study demonstrates the ongoing public health threat of water-related pathogens across Florida. Drivers of waterborne disease, water-based toxins and vector-borne disease are complex, evolving and encompass many variables which influence their emergence and re-emergence across our landscape. Florida is surrounded by water, centered on tourism, home to diverse communities and groups of people, rich with biodiverse species of plants and animals, and host to a wide-range of climatic and weather events. All of these factors have propelled the high rates of water-related disease over the last 20 years. Our findings illustrate recent historical case density and highlight areas for targeted prevention and control efforts.

Overall, the combined cases of salmonellosis, campylobacteriosis, shigellosis, and giardiasis accounted for 87% of the total water-related infections over the last two decades (n = 186,927). Exposure to these waterborne enteric diseases typically occurs following accidental fecal-oral ingestion from contact with contaminated water, food, hands, physical items, soil, infected animals, and filth flies [23]. Unintentional contact with infectious fecal particles in our environment can be due to improper human waste disposal and treatment, such as faulty sewer lines or leaking septic tanks, or unsafe animal waste management, with agricultural runoff polluting waterways, streams, and drinking water sources [24,25]. Foodborne transmission of diarrheal pathogens can result from contamination at the source or at production, during processing, packaging, and marketing, or at the point of use during preparation or consumption [26]. Contact with contaminated soil through cuts, abrasions, as well as through oral, nasal, or ocular routes can transmit several types of waterborne disease and popular outdoor events such as mud sport competitions, camping, and music festivals can increase exposure risks [27]. Previous outbreaks of waterborne disease in Florida have originated from public outdoor areas, parks (community, amusement, state, and water), camps/cabin settings, hotels and resorts, residential housing complexes and mobile home parks, offices, members-only clubs, child and day care centers, assisted living facilities and nursing homes, private homes, municipalities and neighborhoods, schools, bus stations, sports complexes, beaches, and zoos [18].

Florida also has many competent arbovirus vectors and infections are common among mosquito, human and animal populations [28]. Climate impacts the spatial and seasonal distribution of insect vectors as well as their life cycles, feeding and flight patterns, and transmissibility of disease to human or animal host [29]. Florida currently serves as an optimal habitat for mosquito vectors and arboviruses are expected to rise with increases in temperature, precipitation, urbanization and extreme weather events due to climate change [30]. In addition to autochthonous vector-borne disease, the unintentional importation of live, infected mosquitoes through individual or commercial transportation can also lead to focalized disease transmission within native mosquito populations [31,32]. The majority of malaria and dengue viruses cases recorded in Florida are imported, as evidenced by the percentage of cases acquired outside of the United States in this study. Residents may be bitten while traveling to endemic areas and return home with infection [31].

For the majority of confirmed cases of water-based toxins and toxin-producers in this study, such as ciguatera fish poisoning, neurotoxic shellfish poisoning, and saxitoxin poisoning (paralytic shellfish poisoning), most had their exposure within Florida. Previous work has found an increased medical care burden associated with Florida's red tide events with coastal blooms causing acute and chronic respiratory conditions [33]. Animal morbidity and mortality events are common due to exposure to HABs in fresh, brackish, and saltwater systems [34]. The Centers for Disease Control and Prevention (CDC) developed a One Health Harmful Algal Bloom System (OHHABS) in 2016 for states to volunteer case data related to human animal health events surrounding HABs. Between 2016 and 2018, Florida was one of the 18 states who reported a combined 421 HAB events with 389 cases of human illnesses and 413 cases of animal illness/death [35].

Case density of water-related disease often correlated to county population demographics with more confirmed reports in counties with higher resident populations (i.e. Southeast and Central areas of the state). For example, in 2019, the population of Miami-Dade county was over 2.8 million residents and also had the highest number of all reported cases of disease [36]. The counties with the highest water-related disease counts (Miami-Dade, Broward, Palm Beach, Duval, and Hillsborough) are home to not only large residential populations but also large water bodies of fresh, brackish, and saltwater ecosystems [37]. Several of these counties are also home to popular tourist sites, international airports, and ports for cruise attractions and import and export purposes [38]. Furthermore, these counties experience high temperature and rainfall, which can contribute to the proliferation of many water-related pathogens and vectors [12]. The movement of people, plants, and animals within these regions are likely contributing to the spread of disease [39]. In contrast, the area of Florida colloquially known as the *panhandle* demonstrated the lowest case density of confirmed cases. The least populated counties in the state, LaFayette (8482 residents) and Liberty (8772), are found in this area [36]. These counties are also located in the northern areas of Florida, which may hinder the proliferation of the disease vectors due to a slight temperature decrease during winter months [12]. Additionally, many of the less-populated counties are landlocked. While bodies of water such as a lake can facilitate the lifecycle of mosquitoes, a limited number of people within vector range may also decrease transmission risk [40].

Along with the population dynamics and environmental characteristics, the social and individual factors of age, gender, and behavior may pose additional risk factors for the increasing cases of water-related disease in Florida [41,42]. Salmonellosis, the largest proportion of waterborne diseases in Florida, had the highest number of confirmed cases among residents aged 0–4 years. Florida children under five have been contracting waterborne disease more than double the rate of any other age group at just over 70,000 cases over the last two decades. Poor hygiene in young children, exploratory mouthing behaviors, and under-developed immune systems with less infectious doses needed can put this population at an increased risk for waterborne disease exposure and infection [43,44]. Interactive exposure to water parks, pools, beaches or fountains can infect younger age groups with pathogenic agents like *Giardia* spp. and *Cryptosporidium spp.,* likely spread from one infected child to another after accidental fecal exposures to shared water sources [45]. Older children aged 5–9 years were also at high risk for waterborne disease. Likewise, Floridians 70 years of age and older had increased waterborne disease case rates. Older adults and the immuno-compromised are more prone to medical complications or death from water-related infections as compared to other age groups [43]. Older adults are more vulnerable to gastroenteritis than younger age groups with increasing varied health risk factors (ex. diet, immunity, body system functions, dental hygiene, etc.) [46] While children and toddlers have a higher risk exposure to water-related disease due to communal settings such as daycare, older adults are also at an increased risk for transmission within nursing homes and assisted living facilities if they are not safely managed [47,48].

Water-based toxins and water-related vector-borne diseases appear to infect Floridians at much lower rates than waterborne diseases, but they also demonstrate unique case trends among age groups. These diseases have the greatest burden on 45–49-year olds, a time classified within the “working-age” population of 15–64 [49]. Previous work on ciguatera cases in Florida found that within recreational fishers, the highest incidence was within adults between 20 and 64 years of age [50]. Work exposures to water-related pathogens may be due to occupations in agriculture and animal husbandry, conservation and forestry, veterinary care, sewage and solid waste sorting and treatment, landscaping and greenhouse jobs, road/waterway maintenance, plumbers and cooling system care, and seafood markets and fisheries. [51,52]

The Florida ecosystem consists of many unique animal species such as marine and terrestrial mammals, water birds, reptiles, and amphibians- many of which can help spread disease. Numerous water-related pathogens are zoonotic and have been found in Florida's animal populations during this same time period [53]. For example, the water-related vector-borne diseases of West Nile virus, St. Louis encephalitis virus, Venezuelan equine encephalitis and Eastern equine encephalitis have been found in farmed alligators, crested caracaras, feral pigs, and dolphins [ [54], [55], [56], [57]]. Waterborne pathogens have been found in gopher tortoises with *Salmonella spp.*, coyotes with *Cryptosporidium* spp. and *Giardia canis,* and dogs with *Campylocbacter spp.* [[58], [59], [60]] And water-based toxins and toxin-producers such as *Karenia brevis*, saxitoxin, and ciguatoxin have been found in manatees, pufferfish, and lionfish, respectively [[61], [62], [63]]. Currently the FDOH uses sentinel surveillance of arbovirus disease trends among chickens, horses, and mosquito pools to determine seasonal endemicity, to respond to potential outbreaks threats, and to assist in public health messages and alerts [64]. Recent involvement with the CDC's OHHABS system has also been a successful One Health surveillance project [ 35].

Water-related disease transmission can be connected to risk factors such as age, sex and/or gender, environment, lifestyle and behavior, individual immune status, and underlying medical conditions [41,65]. Understanding case trends and risk factors for transmission among people and animals could help reduce the number of cases in Florida. Water-related disease research, prevention, surveillance, and response efforts in Florida should adopt a holistic framework, like the One Health approach, to understand and address the unique environmental, cultural, behavioral, and zoonotic exposure pathways for the most prevalent waterborne diseases, water-related vector-borne diseases, and water-based toxins and toxin-producers impacting Florida [66]. One Health emphasizes the need for multi-stakeholder collaborations such as those between infectious disease researchers, entomologists, physicians, ecologists, economists, veterinarians, epidemiologists, anthropologists, statisticians, agricultural extension officers, farmers, and more to simultaneously address the needs of people, animals, and the environment [67].

Collaborative case and outbreak surveillance and communication between human and animal health care providers is crucial to create effective and comprehensive prevention and response efforts. Surveillance and reporting of water-related illness can assist with allocation of scant resources towards the disease of highest impact, targeting prevention campaigns towards vulnerable groups, or developing environmental interventions within high-risk areas. Disease surveillance systems are steadily becoming easy to use, cheaper, and functional with real-time data, all of which are critical features for an effective joint One Health tool [68]. Improving the ability to respond and even predict cases and outbreaks among humans or animals can increase public and veterinary health agencies with prevention and mitigation strategies [66,67]. Further analysis on Florida's drivers for water-related disease exposure and transmission such as pathogen seasonality, the relationship between animals, vectors, and humans, risk factor variation by county and state region, environmental conditions and viable reservoirs and case demographics will contribute to assisting in future public health efforts for disease preparedness and prevention and bolstering our health care response [10,12].

Although this study analyzed a large amount of previously unexamined historical and regional data, it is not without limitations. The search was restricted to case frequency reports on the public database managed by the Florida Department of Health. Differences in overall case counts for dengue among the two databases during the study period may be due to weekly real-time updates for diagnostic status in the weekly arbovirus surveillance reports. Weekly arbovirus reports on Zika virus infections was left out of the total case counts for water-related vector-borne disease since it was not possible to determine probable from confirmed cases from the FDOH weekly arbovirus reports available online. In addition, case specific information related to risk factors, geographical location, symptoms, treatment, and disease outcome are identifiable and confidential patient information inaccessible to the researchers. Only confirmed cases that were input into the system between January 1st 1999 to December 31st 2019 were included and cases outside this range were excluded, irrespective of exposure date or illness onset. Moreover, case location was determined by county residence of the case and not necessarily where the patient contracted the disease. Finally, as many of the waterborne diseases has multiple exposure pathways, including contaminated food items, the authors cannot accurately determine the vehicle for transmission for these confirmed cases. Additional case information on where the exposure risk occurred will assist public and veterinary health professionals in determining how best to proceed with an intervention.

## 5 Conclusion

Florida's unique geographical characteristics has enabled the presence of water-related infectious agents and advanced the burden of human disease both yesterday and today. The complex relationships between humans, animals and their shared environments, particularly water sources, demands collaboration between disciplines in order to decrease rates of illness among residents. Zoonotic and reverse zoonotic transmission should be a consideration when implementing prevention strategies and policy. A One Health approach between multidisciplinary agencies aimed at human and veterinary public health is critical to adequately respond to these concerns.

## Declaration of interests

The authors declare that they have no known competing financial interests or personal relationships that could have appeared to influence the work reported in this paper.
